# Higher Prevalence of Adverse Childhood Experiences in Transgender Than in Cisgender Individuals: Results from a Single-Center Observational Study

**DOI:** 10.3390/jcm12134501

**Published:** 2023-07-05

**Authors:** Katharina Feil, David Riedl, Bettina Böttcher, Martin Fuchs, Klaus Kapelari, Sofie Gräßer, Bettina Toth, Astrid Lampe

**Affiliations:** 1 Department of Gynecological Endocrinology and Reproductive Medicine, Medical University of Innsbruck, 6020 Innsbruck, Austriabettina.toth@i-med.ac.at (B.T.); 2 University Hospital of Psychiatry II, Department of Psychiatry, Psychotherapy, Psychosomatics and Medical Psychology, Medical University of Innsbruck, 6020 Innsbruck, Austria; sofie.graesser@gmx.net; 3 Ludwig Boltzmann Institute for Rehabilitation Research, 1140 Vienna, Austria; astrid.lampe@rehabilitation.lbg.ac.at; 4 Department of Child and Adolescent Psychiatry, Medical University of Innsbruck, 6020 Innsbruck, Austria; martin.fuchs@tirol-kliniken.at; 5 Department of Pediatrics I, Medical University of Innsbruck, 6020 Innsbruck, Austria; klaus.kapelari@tirol-kliniken.at; 6 VAMED Rehabilitation Center, 6780 Schruns, Austria

**Keywords:** transgender, adverse childhood experience, mental health, trauma, abuse

## Abstract

Adverse childhood experiences (ACE) have been shown to have a tremendous negative impact on health outcomes later in life. This study presents data on the prevalence of ACEs, psychological distress, and trauma-related symptoms in transgender and gender-diverse (TGD) people compared to cisgender people. TGD adults (*n* = 35) and a matched sample of nonpsychiatric hospital patients (*n* = 35) were surveyed between September 2018 and March 2019. Participants completed the Maltreatment and Abuse Chronology of Exposure Scale to assess ACEs, as well as the Brief Symptom Inventory and the Essener Trauma Inventory to assess psychological distress and trauma-related symptoms. TGD patients reported a higher number of ACEs than cisgender patients (0.7 vs. 2.4; *p* < 0.001; d = 0.94). A total of 28.6% of TGD vs. 5.7% cisgender patients reported four or more ACEs (*p* < 0.001). The most common forms of ACEs were parental abuse (54.3%) and peer abuse (54.3%). No significantly increased prevalence of sexual abuse was found (*p* > 0.05). TGD patients also reported a higher prevalence of depression (48.4% vs. 5.7%, *p* < 0.001), posttraumatic stress disorder symptoms (59.4% vs. 13.8%, *p* < 0.001), and anxiety (58.1% vs. 28.6%, *p* = 0.016). Health care providers should be aware of and assess ACEs, especially in vulnerable groups such as TGD people, and create a safe place through open-minded, affirming care.

## 1. Introduction

The terms “transgender” and gender incongruence describe the discrepancy between the assigned and the experienced gender. If the discrepancy leads to distress, this is referred to as gender dysphoria. In *The Diagnostic and Statistical Manual of Mental Disorders, Fifth Edition* (DSM-V) by the American Psychiatric Association, gender dysphoria is defined as clinically relevant distress caused by the discrepancy between the assigned gender and experienced gender [1]. The spectrum of gender is diverse: both a clearly female or male gender identity, in the sense of a binary gender system, as well as a nonbinary gender identity can be sought. Often, but not always, medical treatment options such as gender-affirming hormone therapy (GAHT) or surgical intervention are desired.

The prevalence of transgender and gender-diverse people (TGD) is currently estimated to be 0.3–4.5% of the general adult population and 2.5–8.4% of the general child and adolescent population [2]. The increasing media presence of the topic contributes decisively to the removal of taboos. This is also reflected in the new classification in the International Statistical Classification of Diseases and Related Health Problems (ICD) 11, which was adopted in May 2019 and came into effect in January 2022 [3]. While the ICD 10 still uses the term “transsexualism”, the ICD 11 includes gender incongruence under “conditions related to sexual health”. This contributes decisively to depathologization.

The presence of gender incongruence leads to enormous distress, and TGD people are more likely to suffer from depression and suicidal thoughts and face increased discrimination [4,5,6,7,8]. Treatment options like GAHT or surgical intervention often result in significant improvements in quality of life [6,9,10,11].

In 1998, Felitti and colleagues published the adverse childhood experiences (ACE) study, which examined the long-term effects of childhood abuse on physical and mental health in adulthood [12]. To this day, their work is considered a landmark study, which has triggered a wave of academic research that focused on the relationship between childhood abuse and health in later life.

The term ACE is an umbrella concept which includes potentially traumatic events in childhood such as abuse, neglect, household challenges, bullying, and community violence that can negatively impact health, well-being, and prosperity throughout life [13]. Research has indicated a clear dose–effect relationship, meaning that a higher number of different ACEs is associated with a substantially increased risk for mental health conditions such as depression and suicide attempts as well as for physical and psychosomatic health problems and early death [12,14,15,16,17,18].

An epidemiological study in the U.S. showed that up to 61% of the general population reported at least one ACE and up to 16% were polyvictimized (≥4 ACEs) [19].

However, it was also shown that prevalence rates for ACEs strongly depend on the sample they are drawn from and may substantially differ between countries and populations [20]. While there is already evidence of higher rates of ACEs in the lesbian, gay, and bisexual (LGB) population [21,22,23,24], information on ACEs in TGD people is sparse. Only a few studies were found that explicitly addressed ACEs in TGD people [25,26,27]. In a sample of lesbian, gay, bisexual, transgender, and other (LGBTQ+) youth, higher levels of ACEs were found in TGD youth compared to cisgender youth [26]. The same was shown in a sample of LGBTQ+ adults [25]. Another study showed high rates of ACEs in transgender adults [27]. Childhood adversities were reported in a majority of adult TGD people [28]. Transphobic ACEs had a high prevalence in young transgender women [29]. Adolescent TGD individuals were found to be more likely to have been abused in childhood compared to cisgender individuals [30].

There are several theories on why TGD people have higher rates of ACEs compared to cisgender people. One theory is that TGD people are less conforming to expectations of gender expression and are therefore at higher risk for childhood abuse [31]. Another theory, the minority stress model, states that sexual minorities experience high rates of distress due to stigmas and discrimination and are therefore more likely to suffer from depression and suicidality [32]. A new theory suggests that not only minority stress, but also insufficient social safety can lead to health disparities [21]. Yet another theory sees gender incongruence as a kind of survival response to childhood trauma [33]. A causal relationship between gender incongruence and ACEs can be assumed, which seems most likely to be multifactorial.

The aim of this study was to assess the prevalence of ACEs in a TGD patient population compared to a cisgender patient population and to provide data on psychological distress and trauma-related symptoms in the same populations.

## 2. Materials and Methods

### 2.1. Sample and Procedure

This study is part of a project dealing with the ‘Influence of Violence on the Physical and Psychological Health of Patients in a Hospital Setting’ [15]. In the project, cross-sectional data of >2500 in- and outpatients of seven departments at the University Hospital of Innsbruck (Austria) were collected between October 2015 and March 2019. Informed consent was obtained from all individual participants included in the study. Data collection took place in waiting areas at the hospital. Patients completed a paper-and-pencil questionnaire in private, in a separate section of the waiting area, wherever possible.

Data for this study were collected at the transgender center of the Clinic for Gynecological Endocrinology and Reproductive Medicine (Medical University of Innsbruck) between September 2018 and March 2019. All TGD patients were diagnosed with gender dysphoria according to the DSM-V and were receiving GAHT. A matched control sample was drawn from the project data set.

The study design was approved by the ethics committee of the Medical University of Innsbruck (AN2015-0175 351/4.18) All procedures performed in studies involving human participants were in accordance with the ethical standards of the institutional and/or national research committee and with the 1964 Helsinki declaration and its later amendments or comparable ethical standards.

### 2.2. Measures

#### Maltreatment and Abuse Chronology of Exposure Scale (MACE)

ACEs were assessed with the German version of the Maltreatment and Abuse Chronology of Exposure Scale (MACE). It consists of 75 items that retrospectively assess the severity of exposure to ten types of maltreatment during each year of childhood and adolescence up to the age of 18 years. For each year separately, participants were asked to endorse whether maltreatment occurred during that particular year of their life or not. Thus, onset and cumulative exposure were measured.

The items were grouped into seven ACE clusters: emotional abuse, physical abuse, neglect, witnessing violence (between parents or towards siblings), physical peer abuse, emotional peer abuse, and sexual abuse. Clinical cut-off values for all subscales are available. The MACE provides an overall severity score and multiplicity score (number of types of maltreatment experienced) and has good test–retest reliability and validity [34,35]. In our sample, a good internal consistency for the MACE total score was found (α = 0.86).

### 2.3. Brief Symptom Inventory

Psychological distress was assessed with the Brief Symptom Inventory (BSI-18). Three subscale scores (depression, anxiety, and somatization) and a global score can be computed from it. In this study, specific cut-offs corresponding to experienced gender were applied for the total score as well as cut-offs corresponding to depression, anxiety, and somatization to define clinically relevant symptomatology, as recommended in the German BSI-18 manual. Good psychometric properties have been reported for the BSI-18 [36,37]. In our sample, a good internal consistency for the BSI-18 total score was found (α = 0.89).

### 2.4. Essener Trauma Inventory (ETI)

To assess trauma-related symptoms, the Essener Trauma Inventory (ETI) was used. The ETI was designed to assess various aspects of traumatic events and allows the classification of posttraumatic disorders according to DSM-IV criteria. The ETI assesses 23 trauma-related symptoms, followed by five items assessing functional impairment. Based on the trauma-related symptoms, a total score (range: 0 to 69) can be calculated, with higher values indicating more trauma-related distress. Values of the ETI total score > 16 indicate a notable distress level and values > 27 indicate a clinically relevant level of posttraumatic stress disorder (PTSD) symptoms. Good psychometric properties have been reported for the ETI [38,39]. In our sample, an excellent internal consistency for the ETI total score was found (α = 0.96).

### 2.5. Statistics

Sample characteristics are expressed with descriptive statistics. Analyses were limited to individuals with complete data relating to ACEs and psychological distress. Differences in prevalence rates of the ACE subtypes between TGD and cisgender participants were compared with χ^2^-tests. Associations between sociodemographic variables and the prevalence of overall ACEs as well as ACE subtypes within the TGD group were investigated using Mann–Whitney U-Tests, analyses of variance (ANOVAs), and χ^2^-tests. If more than 20% of cells had frequencies < 5, Fisher’s exact test was applied. Mean differences of overall ACE scores between TGD and cisgender patients were investigated using independent-samples t-tests. To determine the effect size of this mean difference, Cohens’ d was calculated. Values of d < 0.10 were considered negligible, d = 0.1–0.5 as small, d = 0.5–0.8 as moderate, and d > 0.8 as large effects [40]. *p*-values < 0.05 were considered statistically significant. All statistical analyses were performed with IBM SPSS (v22.0).

## 3. Results

A total of *n* = 35 cisgender and *n* = 35 TGD patients were included in the study. Since a gender- and age-matched cisgender patient sample was drawn, the patients’ ages were 29.5 (+/−2.2) years in both samples. In the cisgender group, 54.3% of patients reported male gender compared to 51.4% in the TGD group. Additionally, five patients (14.3%) in the TGD group defined themselves as nonbinary. No significant differences were observed in terms of relationship status, level of education, or living environment. However, TGD patients were significantly less frequently a parent than cisgender patients (5.7% vs. 28.6%, *p* < 0.001). For details see Table 1.

### 3.1. Prevalence of ACEs

Overall, TGD patients reported a significantly higher number of ACEs than cisgender patients (0.7 vs. 2.4; t = 4.172, *p* < 0.001; d = 0.94). In the TGD group, 20.0% (*n* = 7) of the patients reported no ACEs at all, while 54.4% (*n* = 18) reported one to three ACEs and 28.6% reported four or more ACEs (i.e., polytraumatization). This proportion was substantially different in the cisgender group with 65.7% (*n* = 23) reporting no ACEs, 28.6% (*n* = 10) reporting one to three ACEs, and 5.7% (*n* = 2) reporting four or more ACEs (χ^2^ = 16.152, *p* < 0.001).

**Figure 1:**
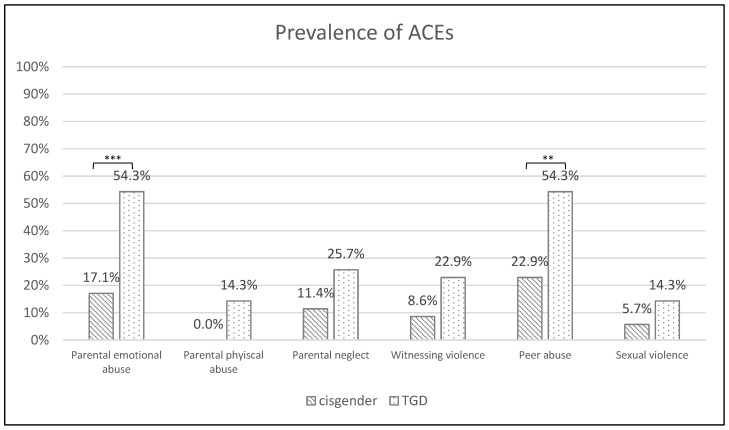
Prevalence rates of adverse childhood experiences (ACEs) in cisgender and TGD patients. *** *p* < 0.001, ** *p* < 0.01.

When comparing the specific types of ACEs between the TGD and cisgender patients, statistically significant differences were found for two ACE forms: TGD patients reported a significantly higher prevalence of parental emotional abuse (54.3% vs. 17.1%; χ^2^ = 10.516, *p* = 0.001) and peer abuse (54.3% vs. 22.9%; χ^2^ = 7.295, *p* = 0.007). No statistically significant differences were observed for parental physical abuse (*p* = 0.054), parental neglect (*p* = 0.22), witnessing intrafamilial violence (*p* = 0.19), or experiencing sexual violence (*p* = 0.21). For details, see Figure 1.

**Figure 2:**
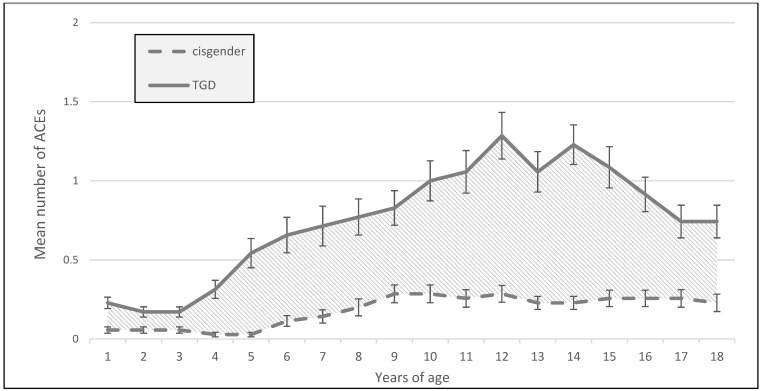
Mean number of adverse childhood experiences (ACEs) above the severity cut-off per age year in the TGD and cisgender groups with standard error of the mean (SEM); hatched area marks the difference in experienced ACEs.

As highlighted in Figure 2, a consistently higher mean number of reported ACEs was found for TGD patients compared to cisgender patients from the age of five years on, with peaks between the ages of 12–14 years.

### 3.2. Association of Sociodemographic Variables with ACEs

To investigate the relationship of ACEs with sociodemographic variables, univariate tests were conducted in the sample of TGD patients. However, there was no statistically significant difference for sex (F(2.34) = 1.545, *p* = 0.23), relationship status (F(2.31) = 0.915, *p* = 0.41), level of education (F(4.32) = 1.576, *p* = 0.21), nor living environment (U = 106.5, *p* = 0.51).

### 3.3. Psychological Distress and Trauma-Related Symptoms

**Figure 3:**
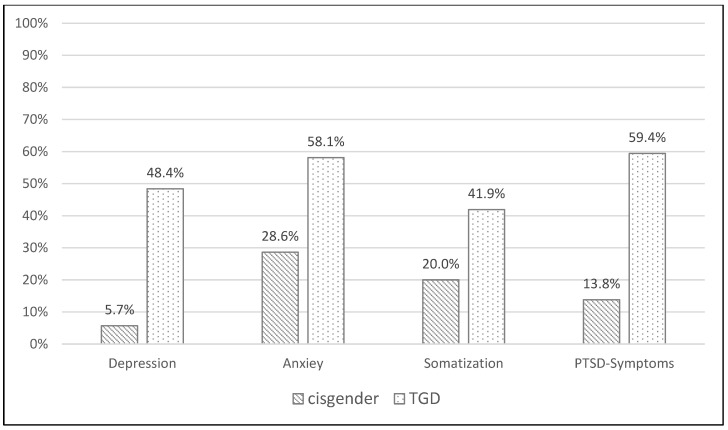
Prevalence rates of depression, anxiety, and somatization (assessed by the Brief-Symptom Inventory; BSI-18) as well as PTSD symptoms (assessed by the Essener Trauma Inventory, ETI) for TGD and cisgender patients.

Overall, a substantially larger proportion of patients with values above the cut-off on the BSI-18 and ETI were found in the sample of TGD patients compared to that of cisgender patients: TGD patients reported a significantly larger prevalence of depression (χ^2^ = 15.654, *p* < 0.001), PTSD symptoms (χ^2^ = 13.457, *p* < 0.001), and anxiety (χ^2^ = 5.854, *p* = 0.016). While prevalence rates were also higher for somatization, this difference was not statistically significant (*p* = 0.053). For details, see Figure 3.

## 4. Discussion

The aim of this study was to present data on the prevalence of ACEs, psychological distress, and trauma-related symptoms in TGD patients compared to cisgender patients. In our sample, the TGD patients reported a significantly higher overall number of ACEs than the cisgender patients. We observed an increase in reported ACEs as early as the age of five years, with a peak between 12–14 years of age.

The percentage of patients who reported to have experienced at least one ACE was more than twice as high in TGD patients than in cisgender patients (80% vs. 34%). Furthermore, in our sample, the number of polytraumatized patients was almost five times higher in TGD patients than in cisgender patients (29% vs. 6%). Previous studies have reported similar prevalence rates:

Craig et al. found significantly higher scores of ACEs in TGD youth (ages 14–18) than in cisgender youth in a LGBTQ+ sample, with 43% of participants reporting four or more ACEs [26]. This was also true in a population of transgender male adults, with 91.6% having experienced at least one and 45% having experienced four or more ACEs [27]. Focusing on LGBTQ+ people, Schnarrs et al. showed that 91.2% of TGD people had experienced at least one ACE and 59.8% had experienced four or more [25]. Biedermann et al. also reported a high prevalence of childhood adversities in TGD people, with 93% having experienced at least one adversity [28]. Two of those studies used cisgender people of sexual minorities as the control group [25,26], while two studies had no control group [27,28]. In addition, Biedermann et al. used the term “childhood adversities” rather than “ACE” and divided the severity of maltreatment into “none to mild”, “mild to moderate”, “moderate to severe”, and “severe to extreme”, which made comparison with the other studies cited difficult [28].

To our knowledge, our study is the first to compare the prevalence of ACEs in TGD people receiving GAHT with that in cisgender people in a representative population of hospital patients. The prevalence rates of ACEs for cisgender patients in our sample was lower than in other studies conducted in the U.S. general population [12,19], while being comparable with studies in the German general population [41,42]. This may be partially caused by different definitions of ACEs; in the initial studies, parental divorce was included in the definition of ACEs, while later on, it was not routinely included. On the other hand, other forms of ACEs, such as peer abuse [16], were newly identified as important ACEs.

TGD patients in our study reported a significantly higher prevalence of parental emotional abuse and peer abuse than cisgender patients. There was no difference between TGD females or males. Previous studies on ACEs in sexual minorities showed higher rates of emotional abuse, emotional neglect, and physical neglect in TGD respondents than in cisgender LGB respondents [25]. Craig et al. found high rates of emotional abuse and neglect in LGBTQ+ youth but did not differentiate between transgender and cisgender youth [26]. Psychological abuse (69%), parental mental illness (64%), divorce of parents (44%), and psychological abuse (40%) were reported in TGD males and most frequently co-occurred with other ACEs [27]. Compared to cisgender youth, physical (odds ratio = 1.61) and psychological abuse (odds ratio = 1.84) were more common in TGD youth [30].

In line with previous studies [25,26,27], TGD patients did not report sexual abuse more frequently than cisgender patients in our sample. Thoma et al., on the other hand, reported higher odds of sexual abuse in TGD adolescents than in cisgender, heterosexual adolescents (odds ratio = 2.04) [30].

It has been highlighted before that TGD adolescents are frequently confronted with emotional and physical peer abuse [43]. Adolescents for whom their heterosexual identity is a key pillar of their personal identity tend to perpetrate more bullying against TGD peers [44]. A recent study in a large Finnish cohort of students found TGD students to be associated with both being bullied and perpetrating bullying (i.e., peer abuse) more frequently than cisgender students. The authors argue that while TGD students are often victimized by peers, perpetration of peer abuse during adolescence may serve as a mechanism to maintain heteronormativity [45]. Thus, the deconstruction of heteronormativity may decrease peer abuse both directed toward TGDs as well as perpetrated by TGDs.

Minority stress or prejudice-related events such as violent attacks or familial rejection can lead to PTSD in sexual minorities [46]. The minority stress model describes how members of minority groups are subjected to chronic stress due to stigmatization [32]. Minority stressors in gay men were described as internalized homophobia, expectations of discrimination and rejection, and actual experience of discrimination and violence [32].

In this study, TGD people reported a significantly higher prevalence of depression, PTSD symptoms, and anxiety than cisgender people. The prevalence rates of TGD patients for anxiety and depression were comparable to those of patients who took part in an outpatient psychotherapy treatment [47] and were substantially higher than in the Austrian general population [48]. This aligns with previous studies, which showed that TGD people who experienced four or more ACEs had five times the odds of reporting depression, suicidality, and PTSD symptoms [27]. Biedermann et al. found that childhood adversities, specifically, emotional and sexual abuse as well as being forced to behave according to the sex assigned at birth, were associated with depression and suicidality in TGD adults [28]. The higher the ACE score, the higher the risk of depression in general [49,50,51]. Experiencing ACEs at a younger age leads to more severe symptoms of PTSD and depression [52].

In this study, an early onset of ACEs, starting at the age of five years, was shown as well as a peak at the age of 12–14 years. A previous study showed a peak of ACEs between 10 and 14 years with a steep increase from the age of 5 years in hospital patients with four or more ACEs [15]. Roberts et al. identified gender nonconformity before the age of 11 years as a risk factor for abuse and probable PTSD [53]. Parental or peer abuse may be directly related to gender-nonconforming behavior. Children and adolescents who feel or behave differently may be more vulnerable to parental or peer abuse, and dysfunctional families may give less parental support to their children [26]. A longer period between realization of one’s gender identity and the onset of transition in TGD individuals has been associated with a longer history of non-suicidal self-injury [54], further underscoring the negative impact of gender dysphoria on mental health. Data on the process of coming out in TGD people are sparse, and no studies could be found on the age of coming out in TGD people associated with ACEs.

### Strengths and Limitations

This study has several strengths and limitations. This is one of the first studies to explicitly investigate a broad range of ACEs in TGD individuals and to compare them with those of a sample of age- and sex-matched cisgender individuals. In comparison to previous studies, which had compared ACEs in TGD individuals mainly to those in other sexual minorities or did not use a control group at all, our findings allow a greater generalizability. An additional strength of our study is the inclusion of peer abuse as a form of ACEs, which has been often neglected in previous ACE studies. The applied questionnaire also allowed participants to retrospectively evaluate the timing of the ACEs, which facilitates the identification of vulnerable periods in the adolescents’ development.

The major limitation is that due to the observational retrospective design of our study, the data do not allow us to test the directionality of the relationship between ACEs and health impairments. While the results strongly indicate that being TGD in a heteronormative society leads to victimization, which in turn has detrimental effects on mental health, our data do not allow a causal interpretation of this relationship. Future studies should use methods that can test for causality. A study with international participation, especially from countries with different acceptance levels of gender incongruence, would be of particular interest here. Additionally, the retrospective assessment of ACEs has to be considered a limitation. A recent meta-analysis found only limited agreement between retrospective and prospective reports of ACEs [55]. While the authors of the meta-analysis argue that retrospective assessment may be more sensitive for ACEs than prospective measures, retrospectively reported exposure is always prone to memory bias. In general, researchers assume that retrospective self-reporting leads to an underestimation of child maltreatment [56].

The sample which was included in our sample was relatively small and collected in as single center, which limits the generalizability of our findings. To avoid statistical issues based on the small sample size, we only applied non-parametric tests for the analyses within the TGD sample. The included sample was an unfiltered ad hoc sample of patients treated at our transgender center and is therefore considered to be somehow representative of TGD patients treated in western Austria. However, the results should be interpreted with caution and we urge for larger international studies to verify the findings from our study.

## 5. Conclusions

In summary, TGD people are significantly more likely to experience ACEs than cisgender people. Parental and peer abuse are especially common among TGD people, while sexual abuse is not more prevalent than in cisgender people. TGD people report a significantly earlier onset of ACEs at five years of age and a significantly higher prevalence of depression, PTSD symptoms, and anxiety than cisgender people. There is the urgent need to prevent childhood trauma in the first place and to strengthen the development of resilience [13]. Health care providers should assess ACEs, especially in vulnerable groups such as TGD people, and create a safe place through open-minded, affirming care.

## Figures and Tables

**Table 1:** Sociodemographic data for cisgender patients and for transgender and gender-diverse patients.

	Cisgender (*n* = 35)		Transgender and Gender Diverse Patients (*n* = 35)			
	Mean	(SD)	Mean	(SD)	t-Value	*p*-Value
Mean age (SD)	29.5	(2.2)	29.5	(2.2)	0.00	1.00
	*n*	%	*n*	%	χ^2^	*p*-value
Gender					5.60	0.06
Male/transgender male	19	54.3%	18	51.4%		
Female/transgender female	16	45.7%	12	34.3%		
Nonbinary	-	-	5	14.3%		
Relationship status					2.67	0.45
Married/long-term relationship	16	48.5%	11	34.4%		
Single	14	42.4%	19	59.4%		
Divorced	2	6.1%	2	6.3%		
Widowed	1	3.0%	0	0.0%		
Missing data	2	5.7%	3	8.6%		
Parenthood	10	28.6%	2	5.7%	63.33	<0.001
Level of education					2.67	0.45
School not finished	1	3.6%	4	12.1%		
Compulsory school	3	10.7%	6	18.2%		
Compulsory school and apprenticeship	12	42.9%	14	42.4%		
Higher education	6	21.4%	7	21.2%		
University degree	6	21.4%	2	6.1%		
Missing data	7	20.0%	2	5.7%		
Living environment					0.59	0.45
Rural	17	48.6%	19	54.3%		
Urban	17	48.6%	13	37.1%		
Missing data	1	2.9%	3	8.6%		

SD = standard deviation.

## Data Availability

The datasets used and/or analyzed during the current study are available from the corresponding author upon reasonable request.
